# Visfatin Promotes IL-6 and TNF-α Production in Human Synovial Fibroblasts by Repressing miR-199a-5p through ERK, p38 and JNK Signaling Pathways

**DOI:** 10.3390/ijms19010190

**Published:** 2018-01-08

**Authors:** Min-Huan Wu, Chun-Hao Tsai, Yuan-Li Huang, Yi-Chin Fong, Chih-Hsin Tang

**Affiliations:** 1 Physical Education Office, Tunghai University, Taichung 40704, Taiwan; mhwu@thu.edu.tw; 2 Sports Recreation and Health Management Continuing Studies, Tunghai University, Taichung 40704, Taiwan; 3 School of Medicine, China Medical University, Taichung 40402, Taiwan; ritsai8615@gmail.com; 4 Department of Orthopedic Surgery, China Medical University Hospital, Taichung 40402, Taiwan; 5 Department of Biotechnology, College of Health Science, Asia University, Taichung 41354, Taiwan; yuanli@asia.edu.tw; 6 Department of Sports Medicine, College of Health Care, China Medical University, Taichung 40402, Taiwan; yichin.fong@msa.hinet.net; 7 Department of Orthopaedic Surgery, China Medical University Beigang Hospital, Yun-Lin County 65152, Taiwan; 8 Graduate Institute of Basic Medical Science, China Medical University, Taichung 40402, Taiwan

**Keywords:** visfatin, IL-6, TNF-α, osteoarthritis, miR-199a-5p

## Abstract

Osteoarthritis (OA), an inflammatory form of arthritis, is characterized by synovial inflammation and cartilage destruction largely influenced by two key proinflammatory cytokines—interleukin-6 (IL-6) and tumor necrosis factor α (TNF-α). Notably, levels of visfatin (a proinflammatory adipokine) are elevated in patients with OA, although the relationship of visfatin to IL-6 and TNF-α expression in OA pathogenesis has been unclear. In this study, visfatin enhanced the expression of IL-6 and TNF-α in human OA synovial fibroblasts (OASFs) in a concentration-dependent manner and stimulation of OASFs with visfatin promoted phosphorylation of extracellular-signal-regulated kinase (ERK), p38, and c-Jun N-terminal kinase (JNK), while ERK, p38, and JNK inhibitors or siRNAs all abolished visfatin-induced increases in IL-6 and TNF-α production. Moreover, transfection with miR-199a-5p mimics reversed visfatin-induced increases in IL-6 and TNF-α production. Furthermore, we also found that visfatin-promoted IL-6 and TNF-α production is mediated via the inhibition of miR-199a-5p expression through the ERK, p38, and JNK signaling pathways. Visfatin may therefore be an appropriate target for drug intervention in OA treatment.

## 1. Introduction

Osteoarthritis (OA), a common chronic inflammatory disorder of the synovial joint, is characterized by articular cartilage degradation, cartilage remodeling/degeneration, subchondral sclerosis and osteophyte formation [1,2,3]. OA patients typically complain of stiffness, muscle weakness and joint pain; the etiology underlying these symptoms remains unclear [4,5]. It is known that an inflammatory reaction promotes the overexpression of macrophage-derived proinflammatory cytokines interleukin 1 β (IL-1β), IL-6 and tumor necrosis factor α (TNF-α) in the synovial membrane, which induces neovascularization and inflammation as well as the production of matrix-degrading enzymes such as matrix metalloproteinases (MMPs) that lead to cartilage degradation [6,7].

IL-6 involves several biological functions and plays a key role in hematopoiesis formation [8], the innate immune response, inflammation and promotes osteoclastogenesis which is recognized as being a critical proinflammatory cytokine in the pathophysiology of OA [9]. Another key proinflammatory cytokine, TNF-α is a potent stimulus of inflammatory responses through the up-regulation of several genes, including those responsible for cytokines, chemokines, proteinases, cyclooxygenase, and adhesion molecules [10,11]. Notably, elevated TNF-α levels are found in human OA synovial fluid [12,13].

Visfatin, a growth factor for B lymphocyte precursors, is a pro-inflammatory adipokine found in the liver, skeletal muscles and bone marrow and produced by visceral white adipose tissue, which mimics the effects of insulin [14]. Serum visfatin levels are increased in patients with OA [15,16]. Reports have highlighted the significant role played by adipocytokines, including visfatin, in mediating joint damage [17,18]. However, the role of visfatin in IL-6 and TNF-α production in osteoarthritis synovial fibroblasts (OASFs) has not been extensively studied. We therefore sought to elucidate the intracellular signaling underlying visfatin-induced IL-6 and TNF-α production in human OASF cells. Our findings show that visfatin promotes IL-6 and TNF-α production by repressing miR-199a-5p expression via the extracellular-signal-regulated kinase (ERK), p38 and c-Jun N-terminal kinase (JNK) signaling pathways. Thus, we suggest that visfatin could be an appropriate target for therapeutic intervention in OA.

## 2. Results

### 2.1. Visfatin Promotes IL-6 and TNF-α Expression in Human Osteoarthritis Synovial Fibroblasts (OASFs)

**Figure 1:**
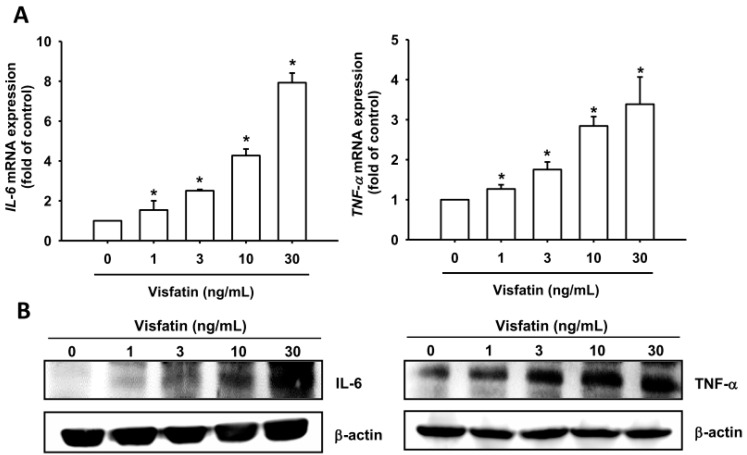

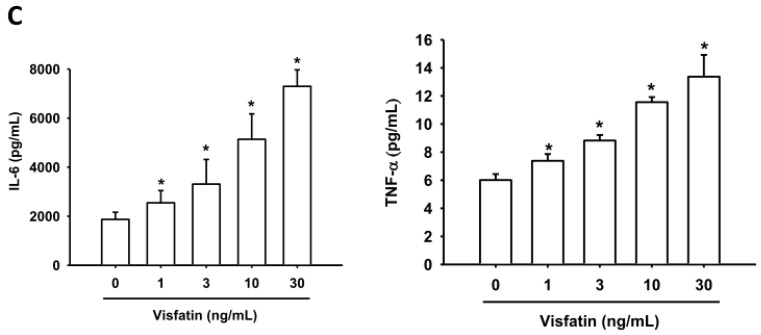
Visfatin induces IL-6 and TNF-α expression in human synovial fibroblasts. Osteoarthritis synovial fibroblasts (OASFs) were incubated with various concentrations of visfatin for 24 h. (**A**–**C**) IL-6 and TNF-α expression was examined by qPCR, Western blot and ELISA assay. Results are expressed as the mean ± SEM. * *p* < 0.05 as compared with baseline.

Visfatin levels are significantly higher in synovial fluid from patients with OA compared with healthy controls [17,18]. OA pathology is associated with chronically inflamed synovium, increased levels of inflammatory cells and synovial hyperplasia, as well as fibroblast-like synoviocytes [19]. We therefore used human synovial fibroblasts to investigate which signaling pathways involve visfatin in the production of IL-6 and TNF-α. Treatment of OASFs with visfatin (1–30 ng/mg) for 24 h induced *IL-6* and *TNF-α* mRNA expression in a concentration-dependent manner (Figure 1A). Visfatin also enhanced the protein expression of IL-6 and TNF-α according to Western blot and ELISA analysis (Figure 1B,C). These results indicate that visfatin enhances IL-6 and TNF-α expression in human OASFs.

### 2.2. Visfatin Increases IL-6 and TNF-α Expression via the MAPK Signaling Pathway

**Figure 2:**
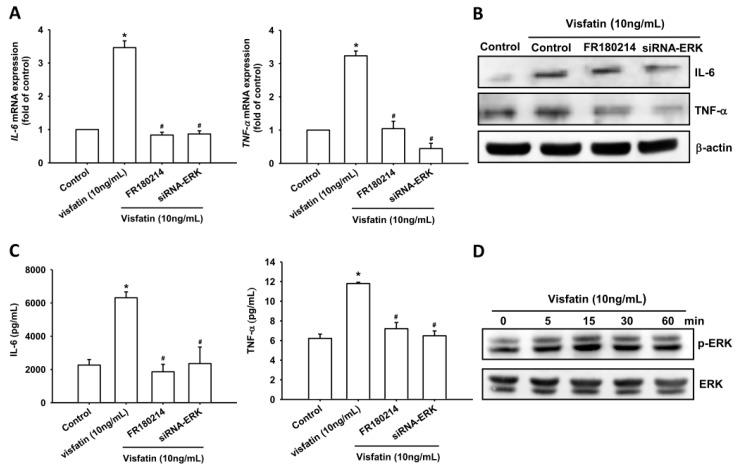
Visfatin induces increases in IL-6 and TNF-α expression through the ERK pathway. (**A**–**C**) OASFs were pretreated with FR180214 (10 μM) for 30 min or transfected with ERK siRNA for 24 h followed by stimulation with visfatin (30 ng/mL) for 24 h; IL-6 and TNF-α expression was examined by qPCR, Western blot and ELISA assay; (**D**) OASFs were incubated with visfatin for indicated time intervals; ERK phosphorylation was examined by Western blot. Results are expressed as the mean ± SEM. * *p* < 0.05 as compared with baseline. ^#^ *p* < 0.05 as compared with the visfatin-treated group.

**Figure 3:**
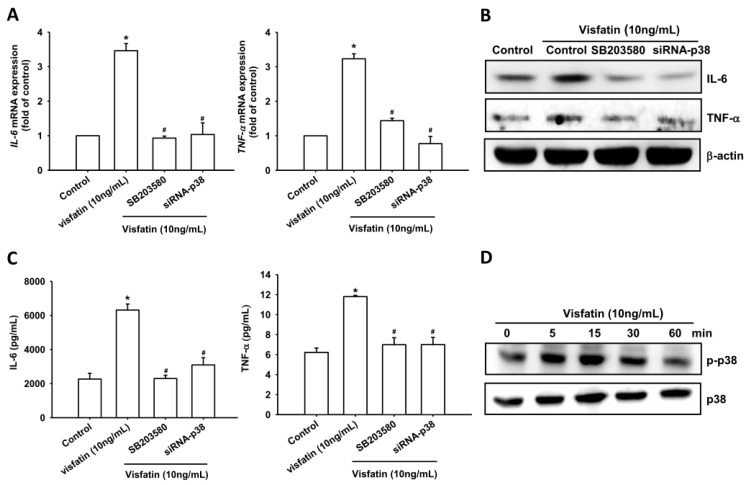
Visfatin induces increases in IL-6 and TNF-α expression through the p38 pathway. (**A**–**C**) OASFs were pretreated with SB203580 (10 μM) for 30 min or transfected with p38 siRNA for 24 h followed by stimulation with visfatin (30 ng/mL) for 24 h; IL-6 and TNF-α expression was examined by qPCR, Western blot and ELISA assay; (**D**) OASFs were incubated with visfatin for indicated time intervals; p38 phosphorylation was examined by Western blot. Results are expressed as the mean ± S.E.M. * *p* < 0.05 as compared with baseline. ^#^ *p* < 0.05 as compared with the visfatin-treated group.

**Figure 4:**
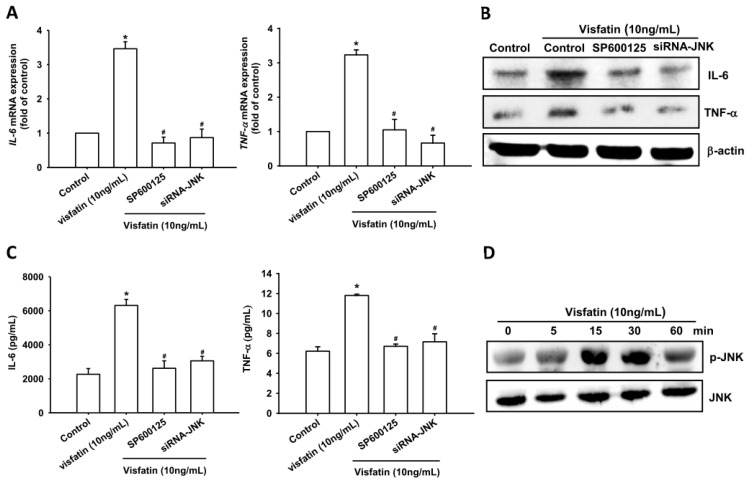
Visfatin induces increases in IL-6 and TNF-α expression through the JNK pathway. (**A**–**C**) OASFs were pretreated with SP600125 (10 μM) for 30 min or transfected with JNK siRNA for 24 h followed by stimulation with visfatin (30 ng/mL) for 24 h; IL-6 and TNF-α expression was examined by qPCR, Western blot and ELISA assay; (**D**) OASFs were incubated with visfatin for indicated time intervals; JNK phosphorylation was examined by Western blot. Results are expressed as the mean ± SEM. * *p* < 0.05 as compared with baseline. ^#^ *p* < 0.05 as compared with the visfatin-treated group.

Previous studies have shown that the mitogen-activated protein kinases (MAPKs), ERK, p38 MAPK and JNK are involved in the regulation of inflammatory cytokine expression [20,21]. We therefore investigated the role of MAPKs in mediating visfatin-induced IL-6 and TNF-α expression, using the specific ERK inhibitor FR180214, p38 inhibitor SB203580, and JNK inhibitor SP600125. Pretreatment of OASFs with these agents blocked visfatin-induced increases in mRNA expression of *IL-6* and *TNF-α* levels (Figure 2A–C, Figure 3A–C and Figure 4A–C). In addition, transfection of OASFs with ERK, p38 and JNK siRNAs markedly inhibited visfatin-enhanced IL-6 and TNF-α production (Figure 2A–C, Figure 3A–C and Figure 4A–C), whereas incubation of OASFs with visfatin promoted ERK, p38 and JNK phosphorylation in a time-dependent manner (Figure 2D, Figure 3D and Figure 4D). Thus, visfatin appears to act through the MAPK signaling pathway to promote IL-6 and TNF-α expression in OASFs.

### 2.3. Visfatin Increases IL-6 and TNF-α Production in OASFs by Inhibiting miR-199a-5p Expression

**Figure 5:**
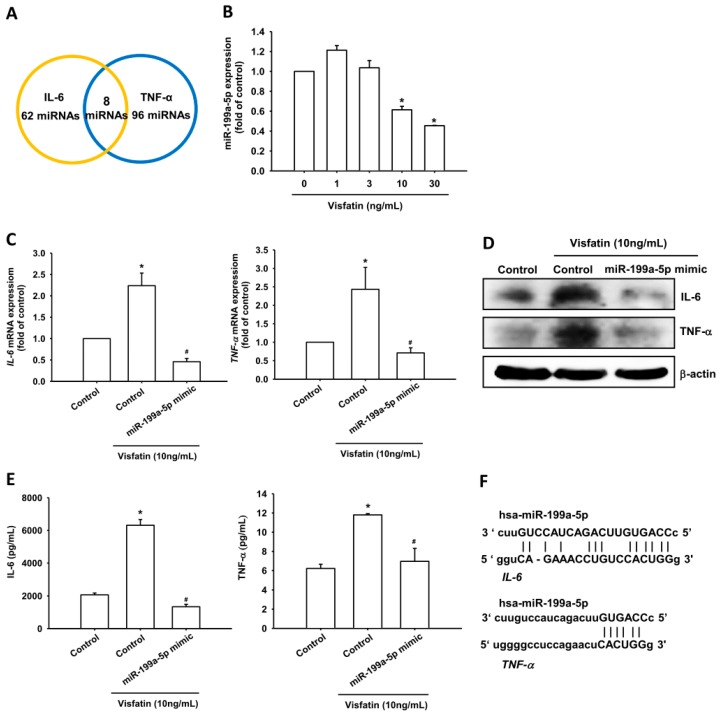

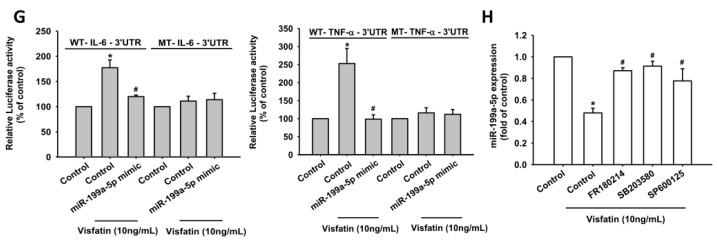
Visfatin increases IL-6 and TNF-α expression via inhibition of miR-199a-5p through the ERK, p38 and JNK signaling pathways. (**A**) Searches of three online computational algorithms (TargetScan, miRWalk and miRanda) for candidate miRNAs that target the IL-6 and TNF-α regions revealed the involvement of miR-199a-5p; (**B**) OASFs were incubated with visfatin for 24 h; miR-199a-5p expression was assessed by qPCR; (**C**–**E**) OASFs were transfected with miR-199a-5p mimic for 24 h, followed by stimulation with visfatin for 24 h; IL-6 and TNF-α expression was examined by qPCR, Western blot and ELISA assay; (**F**) Schematic 3′-UTR representation of human *IL-6* and *TNF-α* containing the miR-199a-5p binding site; (**G**) OASFs were transfected with indicated luciferase plasmids before incubation with visfatin for 24 h; Luciferase activity was assessed; (**H**) OASFs were pretreated with ERK, p38, and JNK inhibitors for 30 min followed by stimulation with visfatin (30 ng/mL) for 24 h; miR-199a-5p expression was examined by qPCR. Results are expressed as the mean ± SEM. * *p* < 0.05 as compared with baseline. ^#^ *p* < 0.05 as compared with the visfatin-treated group.

miRNAs are important regulators of inflammatory cytokine production [22,23] and have recently been implicated in the control of OA pathogenesis [24,25,26]. We therefore hypothesized that miRNAs may regulate visfatin-mediated IL-6 and TNF-α expression. Using miRNA target prediction software, we found that the 3′-UTRs of *IL-6* and *TNF-α* mRNAs harbor potential binding sites for miR-199a-5p (Figure 5A). Stimulation of OASFs with visfatin lowered miR-199a-5p expression in a concentration-dependent manner (Figure 5B). Further investigations confirmed the involvement of miR-199a-5p in visfatin-induced increases in IL-6 and TNF-α mRNA and protein expression; miR-199a-5p mimic reversed these increases (Figure 5C–E). Our data suggest that visfatin increases IL-6 and TNF-α production by inhibiting miR-199a-5p expression.

To learn whether miR-199a-5p regulates the 3′-UTRs of *IL-6* and *TNF-α*, we constructed luciferase reporter vectors harboring the wild-type 3′-UTRs of *IL-6* and *TNF-α* mRNAs (IL-6-3′-UTR-wt and TNF-α-3′-UTR-wt) and a vector containing mismatches in the predicted miR-199a-5p binding sites (IL-6-3′-UTR-mut and TNF-α-3′-UTR-mut) (Figure 5F). We found that transfection with the miR-199a-5p mimic antagonized visfatin-induced increases in luciferase activity in the IL-6-3′-UTR-wt and TNF-α-3′-UTR-wt but not in the IL-6-3′-UTR-mut and TNF-α-3′-UTR-mut plasmids (Figure 5G). In addition, treatment with ERK, p38 and JNK inhibitors reversed visfatin-mediated miR-199a-5p expression (Figure 5H). Collectively, these data suggest that miR-199a-5p directly represses IL-6 and TNF-α expression via binding to the 3′-UTR region of the human *IL-6* and *TNF-α* genes through the ERK, p38 and JNK pathways.

## 3. Discussion

It is well established that a complex cytokine network contributes to chronic inflammation of the synovial membrane and the development of disease and cartilage degradation in OA [27]. However, we lack a complete understanding of what factors are responsible for initiating the degradation and loss of articular tissue. While elevated levels of the proinflammatory adipokine, visfatin, are observed in inflammatory diseases, such as OA and rheumatoid arthritis (RA) [28], the molecular mechanisms regulating this inflammatory response are unclear. In this study, we demonstrated that IL-6 and TNF-α are target proteins for the visfatin signaling pathway regulating the cell inflammatory response. Furthermore, we found that visfatin enhances IL-6 and TNF-α production by inhibiting miR-199a-5p via the ERK, p38 and JNK signaling pathways in OASFs. These findings suggest that visfatin enhances proinflammatory cytokines, such as IL-6 and TNF-α, and the inflammatory response. IL-1β is another key proinflammatory cytokine that enhances the production of MMPs leading to cartilage degradation [6,7]. However, we did not examine the role of visfatin in IL-1β and MMPs production in OASFs; this aspect needs further analysis. It is well established that OA chondrocytes produce higher levels of MMP-1, -3 and -13 in comparison with normal chondrocytes [17,29]. Therefore, chondrocyte activity is also an important factor for progression of OA. However, our study did not investigate the role of visfatin in chondrocyte cells; this is another research area that requires further clarification.

The MAPK signaling pathway helps to regulate gene expression levels [30]. Here, we report that the ERK inhibitor FR180214, p38 inhibitor SB203580, and JNK inhibitor SP600125 all antagonized visfatin-induced increases in IL-6 and TNF-α expression. Similar results were seen after OASFs were transfected with ERK, p38 and JNK siRNAs, while stimulation of OASFs with visfatin promoted ERK, p38, and JNK phosphorylation. These results indicate that ERK, p38, and JNK activation mediates visfatin-promoted IL-6 and TNF-α production in OASFs.

miRNAs have been investigated for their role in gene regulation [31]. By binding to mRNA 3′-UTRs, miRNAs can affect many protein-encoding genes at the post-transcriptional level [32]. We therefore sought to determine whether miRNAs are implicated in IL-6 and TNF-α expression following visfatin stimulation. We found that visfatin markedly inhibits miR-199a-5p expression in OASFs. Co-transfection of cells with miR-199a-5p mimic abolished visfatin-induced increases in IL-6 and TNF-α expression. Strikingly, we found that miR-199a-5p directly inhibited IL-6 and TNF-α protein expression through binding to the 3′-UTRs of the human *IL-6* and *TNF-α* genes, thereby negatively regulating visfatin-mediated IL-6 and TNF-α expression. These findings provide insight into potential miRNA-based strategies for visfatin-mediated IL-6 and TNF-α production.

**Figure 6:**
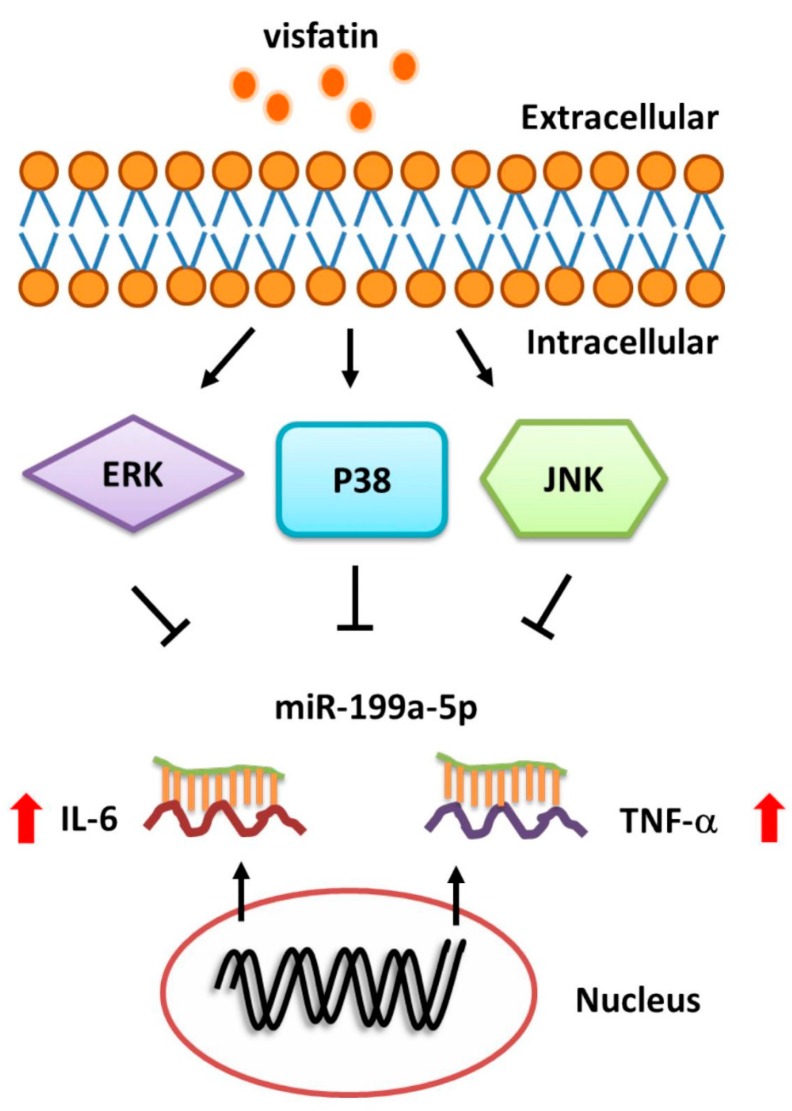
Schema of signaling pathways involved in visfatin-induced increases in IL-6 and TNF-α expression in synovial fibroblasts. Visfatin promotes IL-6 and TNF-α production (red arrows) in human synovial fibroblasts by inhibiting miR-199a-5p expression (T bars) via the ERK, p38 and JNK signaling pathways (black arrows).

In conclusion, our investigations into the signaling pathway involved in visfatin-induced increases in IL-6 and TNF-α expression in human synovial fibroblasts reveal that visfatin inhibits miR-199a-5p expression through the ERK, p38 and JNK signaling pathways (Figure 6). These findings may provide a better understanding of the mechanisms of OA pathogenesis.

## 4. Materials and Methods

### 4.1. Materials

We obtained control miRNA, miR-199a-5p mimic and Lipofectamine 2000 from Life Technologies (Carlsbad, CA, USA), rabbit polyclonal antibodies for P-ERK, ERK, P-p38, p38, P-JNK, JNK, TNF-α, IL-6 and β-actin; ERK, p38, JNK and control siRNA were purchased from Santa Cruz Biotechnology (Santa Cruz, CA, USA), recombinant human visfatin was purchased from PeproTech (Rocky Hill, NJ, USA). All other chemicals were purchased from Sigma-Aldrich (St. Louis, MO, USA).

### 4.2. Cell Culture

Human synovial fibroblasts were obtained from synovial tissue collected from generally healthy OA patients aged 50–75 years undergoing knee replacement surgery. Written approval (CMUH103-REC2-023, 6 May 2014) was obtained from the Institutional Review Board of China Medical University Hospital, Taichung, Taiwan, and also from the patients, prior to sample collection. OASFs were isolated, cultured, and characterized as previously described [33,34]. In vitro experiments were performed using cells from passages 3–6.

### 4.3. Measurement of IL-6 and TNF-α

We pretreated human OASFs with various inhibitors for 30 min or transfected the OASFs for 24 h with miRNA mimic or siRNAs prior to visfatin administration. IL-6 and TNF-α in the medium was assayed using IL-6 and TNF-α enzyme immunoassay kits (R&D Systems, Minneapolis, MN, USA), as according to the manufacturer’s procedure.

### 4.4. Real-Time Quantitative PCR of mRNA and miRNA

Total RNA was extracted from OASFs using a TRIzol kit (MDBio, Taipei, Taiwan). Reverse transcription was performed using oligo(dT) primer [35,36]. We used the Taqman^®^ one-step PCR Master Mix (Applied Biosystems, Foster City, CA, USA) to perform real-time quantitative PCR (RT qPCR) analysis; we added 100 ng of total cDNA per 25 µL reaction with sequence-specific primers and Taqman^®^ probes. We purchased sequences for target gene primers and probes from Applied Biosystems (GAPDH was used as the internal control); qPCR assays were carried out in triplicate using the StepOnePlus sequence detection system (Applied Biosystems, Foster City, CA, USA) [37].

For the miRNA assay, we used the TaqMan MicroRNA Reverse Transcription Kit (Applied Biosystems) to synthesize cDNA; reactions were incubated at 16 °C for 30 min, then at 42 °C for 30 min, followed by inactivation at 85 °C for 5 min. All reactions were run using the StepOnePlus sequence detection system. Relative quantification analysis of gene expression was performed with the *U6* gene as an endogenous control. The relative gene expression level was calculated using the comparative C_T_ method.

### 4.5. Western Blot Analysis

The cell lysates were resolved by SDS-PAGE [7,38] and transferred to Immobilon polyvinylidene fluoride membranes. We initially blocked blots for 1 h with 4% bovine serum albumin at room temperature, then probed the blots with rabbit anti-human antibodies against P-ERK, ERK, P-p38, p38, P-JNK or JNK (1:1000) for 1 h at room temperature (Santa Cruz Biotechnology, Santa Cruz, CA, USA). After undergoing three washes, blots were incubated secondary antibody (1:1000) visualized using LAS-4000 image reader (FujiFILM, Tokyo, Japan) [39].

### 4.6. Plasmid Construction and Luciferase Assay

The three prime untranslated region (3′-UTR) of human *IL-6* and *TNF-α* contains a miR-199a-5p binding site. DNA fragments containing wild-type (wt)-IL-6-3′-UTR, mutant-type (mut)-IL-6-3′-UTR, wt-TNF-α-3′-UTR and mut-TNF-α-3′-UTR were purchased from Invitrogen (Carlsbad, CA, USA). The fragments were subcloned into the luciferase reporter vector pmirGLO-control (Promega, Madison, WI, USA), upstream of the vector’s promoter. These plasmids were transfected into cells using Lipofectamine 2000. Cell extracts were prepared and used to measure luciferase and β-galactosidase activity.

### 4.7. Statistical Analysis

The data are expressed as the mean ± SEM. Statistical analysis was performed using GraphPad Prism 4 software (GraphPad Software, La Jolla, CA, USA). One-way analysis of variance (ANOVA) and the unpaired two-tailed Student’s *t*-test were used to test for any significant difference in the means. The difference was denoted significant when the *p*-value was less than 0.05.
